# Different precipitation response over land and ocean to orbital and greenhouse gas forcing

**DOI:** 10.1038/s41598-020-68346-y

**Published:** 2020-07-17

**Authors:** Chetankumar Jalihal, Jayaraman Srinivasan, Arindam Chakraborty

**Affiliations:** 1 Centre for Atmospheric and Oceanic Sciences, Indian Institute of Science, Bangalore, 560012 India; 2 Divecha Centre for Climate Change, Indian Institute of Science, Bangalore, 560012 India

**Keywords:** Atmospheric dynamics, Palaeoclimate, Climate and Earth system modelling

## Abstract

Various proxies suggest a nearly in-phase variation of monsoons with local summer insolation. Oceanic proxies of monsoons document a more complex response. Climate model simulations also indicate that the response is different over land and ocean. Here using a transient simulation by a climate model over the last 22,000 years we have unraveled the factors that lead to these differences within the Indian subcontinent. We show that during the deglacial (22–12 ka) precipitation over India and the Bay of Bengal (BoB) are in phase, whereas they are out of phase across the Holocene ($\sim$ 12 ka to 0 ka). During the deglacial, water vapor amplifies the effect of solar forcing on precipitation over both the regions, whereas contributions from surface latent heat fluxes over the BoB drive an opposite response across the Holocene. We find that greenhouse gas forcing drives similar precipitation response over land and ocean, whereas orbital forcing produces a different response over land and ocean. We have further demonstrated that during periods of abrupt climate change [such as the Bølling–Allerød ($\sim$ 14 ka)], water vapor affects precipitation mainly through its influence on the vertical stability of the atmosphere. These results highlight the complex nature of precipitation over the BoB and thus has implications for the interpretation of monsoon proxies.

## Introduction

There is substantial proxy-based evidence to suggest that terrestrial precipitation over the northern hemisphere vary in near synchrony with each other and are mainly driven by the variations in solar insolation^1^. This has been attributed to the latitudinal shift in the zonal mean intertropical convergence zone (ITCZ), which is driven by the differential warming of the two hemispheres^2–4^. Recent modeling studies have, however, pointed out that the precipitation over land and ocean respond differently to orbital forcings^5–8^. This is further supported by a 4,000-year speleothem record from the Baratang cave in the Andaman Islands located in the Bay of Bengal (BoB)^9^. The proxy suggests that precipitation increased in the BoB over the last 4,000 years, whereas speleothem records from mainland India show a decrease in monsoon over the same period^10^. This differential response of the land and ocean highlights the role of local factors, which are as important as the changes in solar insolation forcing.

The mechanism for this differential response has been explained with the moist static energy budget^7^ and energetics of monsoon and ITCZ^5, 8,11,12^. The atmosphere over land responds quickly to a change in insolation forcing and acts to export the excess energy out of the column^7^. This leads to a stronger monsoon circulation. The oceans import the excess energy by changing the vertical structure of the atmosphere. Though this explains the mean tropical picture, the mechanisms are more complex for regional monsoons^8^. Over the BoB, the surface latent heat fluxes play an important role in driving orbital scale changes in precipitation. The perturbations in surface latent heat flux are large enough to counter the changes in insolation forcing. These perturbations are driven by winds, which are in turn a response to convective heating over the middle-east and the west equatorial Indian ocean. All the previous studies that advocate this differential response of precipitation focused mainly on the precessional variations in solar insolation, while the other boundary conditions were kept constant corresponding to that of the interglacials. Thus, their conclusions do not reflect the total evolution of this differential response across glacial-interglacial cycles.

In this paper, we have explored the evolution of boreal summer precipitation over land vis-à-vis that over the ocean to realistic transient forcings over the last 22,000 years. Earth transitioned from the colder glacial to the warmer interglacial climate during this period. Hence, it is suitable for our study. We have used results from a transient climate simulation, the TraCE-21k^13,14^. This simulation uses the fully coupled general circulation model CCSM3, forced with realistic transient forcings (orbital parameters, greenhouse gases, ice sheets, and meltwater fluxes). We have used a diagnostic model for moisture convergence over well-defined monsoon regions, based on the vertically integrated moist static energy and moisture budgets to investigate the changes in precipitation (see “Methods” for more details).

## Results

### Orbital scale variations

The peak of the last glacial period known as the Last Glacial Maximum (LGM) occurred around 21,000 years ago^15–17^. Greenhouse gas concentrations were low^18^ and large ice sheets covered North America and Europe^15–17^. Over the period 18–11.6 ka (referred to as the deglacial^19^) boreal summer insolation increased due to precession and reached a maximum towards the end of this period^20^. $\hbox {CO}_{2}$ rose rapidly from 180 to 260 ppm^19^, and ice sheets receded^16,17^. The melting of ice sheets released fresh water into the North Atlantic in abrupt pulses, which led to a slowdown of the Atlantic Meridional Overturning Circulation (AMOC)^21,22^. This triggered large centennial-to-millennial scale oscillations in climate across the planet^1, 2,13,23,24^. During the period 11,600 years ago to present (known as the Holocene)^25^, ice-sheet extent and $\hbox {CO}_{2}$ concentrations remained nearly constant. Insolation was the dominant forcing across the Holocene. The boreal summer insolation declined across the Holocene due to the precession of the Earth^20^.

**Figure 1:**
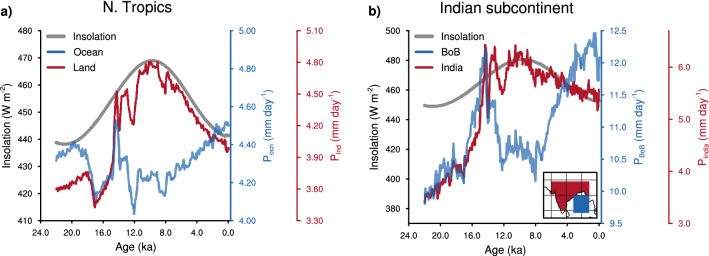
The temporal evolution of precipitation over land and ocean. The time series of Jun–Jul–Aug (JJA) averaged insolation (grey), precipitation over land (red) and precipitation over ocean (blue) for (**a**) northern tropics (0$^\circ$–30$^\circ$ N and 0$^\circ$–360$^\circ$ E), (**b**) the Indian subcontinent. The region considered for the Indian summer monsoon is shown in the inset map in (**b**) (10$^\circ$–29$^\circ$ N and 70$^\circ$–95$^\circ$ E; land only grids), and for the Bay of Bengal the domain is (9$^\circ$–20$^\circ$ N and 85$^\circ$–95$^\circ$ E; ocean only grids).

Shown in Fig. 1a, is the time series of boreal summer precipitation over all the northern tropical land and ocean taken separately in the TraCE-21k. Precipitation over land largely follows the insolation, particularly during the Holocene. Additional forcing from greenhouse gases and ice sheets through most of the deglacial, amplify the effect of insolation^26^. The precipitation over both land and ocean shows a coherent behavior during this period. They have, however, an opposite trend across the warm and stable Holocene. This changing relation between precipitation over land and ocean has never been explored fully before and hence this is the focus of the present work.

**Figure 2:**
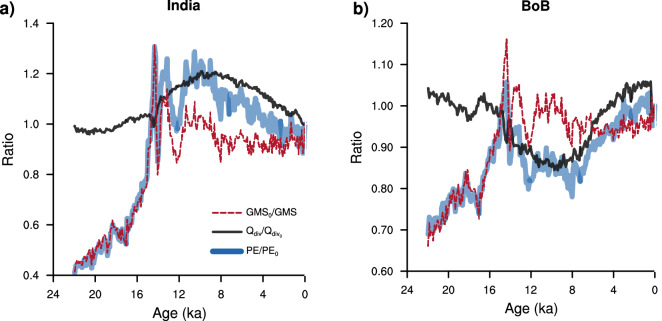
The relative contribution of $Q_{{\mathrm{div}}}$ and *GMS* to $P-E$. Time series of the ratio of moisture convergence ($P-E$) in blue, total energy flux into the atmosphere ($Q_{{\mathrm{div}}}$; sum of top and bottom fluxes) in black, and the gross moist stability (*GMS*) in red with respect to their respective pre-industrial (averaged over 1750–1850 AD) values ($PE_{0}$, $Q_{{\mathrm{div0}}}$, and $GMS_{0}$) for (**a**) India (land grids only), (**b**) the Bay of Bengal (ocean grids only).

This unique response of land and ocean precipitation to glacial-interglacial changes is also observed at a regional scale (Fig. 1b). Within the Indian monsoon system, precipitation over the Indian landmass and the BoB respond in a similar manner to the deglacial forcing, and in a different manner to the Holocene forcing. This is in agreement with the proxies (Supplementary Fig. 1). Precipitation over both the regions increases through most of the deglacial (22–15 ka). Over the period 15–12 ka, precipitation over India continues to increase, whereas that over the BoB decreases even though insolation continues to strengthen during this period. Across the Holocene, following the decline in summer insolation, precipitation over India decreases. There is, however, an increase in precipitation over the BoB during the Holocene. We have used the energetics of monsoons to unravel the factors that lead to a different response of the Indian summer monsoon (ISM; land only grids) and BoB (oceanic grids only) to the external forcing. Our methodology is based on the budgets of vertically integrated moist static energy (*MSE*) and moisture convergence^27^, and is detailed in the “Methods” section. The equation for moisture convergence (i.e rainfall–evaporation, $P-E$; units—mm $\hbox {day}^{-1}$) is:

1 $$\begin{aligned} P - E = \frac{Q_{div}}{GMS} \end{aligned}$$

where $Q_{{\mathrm{div}}}$ is the sum of all the energy fluxes going into the atmosphere (units—mm $\hbox {day}^{-1}$; 28.9 W $\hbox {m}^{-2} =$ 1 mm $\hbox {day}^{-1}$). Over land, since the net surface fluxes are small, $Q_{{\mathrm{div}}}$ essentially represents the net downward radiative fluxes at the top of the atmosphere. The gross moist stability (*GMS*) denotes the net lateral export of *MSE*^28^. *GMS* amplifies or dampens the effect of $Q_{{\mathrm{div}}}$ on $P-E$. It depends predominantly on the vertical profiles of *MSE*, specific humidity, and vertical velocity^29^. The form of the equation is identical to that derived earlier^28^. Figure 2 depicts the relative dominance of $Q_{{\mathrm{div}}}$ and *GMS* during different periods for the ISM (Fig. 2a) and BoB (Fig. 2b). $P-E$ over both the regions have a distinct *GMS* dominant regime ($\sim$ 22 ka to 12 ka) and a $Q_{{\mathrm{div}}}$ dominant regime ($\sim$ 12 ka to present). During the *GMS* dominant regime, $P-E$ over both the land and ocean grids show similar trends, whereas they exhibit an opposite trend during the $Q_{{\mathrm{div}}}$ dominant regime. To understand this further, we look at the evolution of the $Q_{{\mathrm{div}}}$ and *GMS* over the last 22,000 years for both the regions.

**Figure 3:**
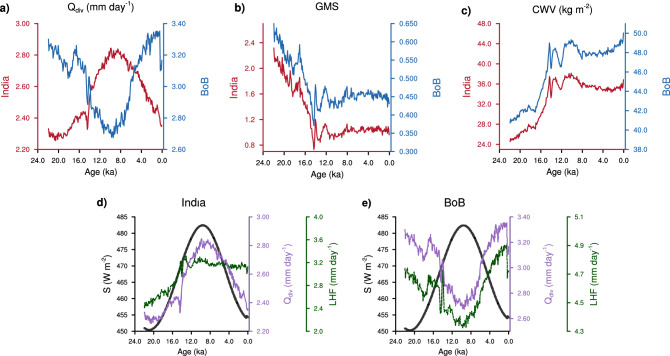
The phase of $Q_{{\mathrm{div}}}$ and *GMS* between India and BoB. Time series of (**a**) total energy flux into the atmosphere ($Q_{{\mathrm{div}}}$; top $+$ bottom), (**b**) *GMS*, and (**c**) column integrated water vapor (*CWV*), for India (red) and the Bay of Bengal (blue). (**d**, **e**) show the decomposition of $Q_{{\mathrm{div}}}$ into its components: insolation (black), $Q_{{\mathrm{div}}}$ (purple), and surface latent heat flux (green) over India and the Bay of Bengal, respectively.

$Q_{{\mathrm{div}}}$ over India and the BoB are in general out of phase with each other (Fig. 3a). $Q_{{\mathrm{div}}}$ over India starts with low values at LGM and then increases across the deglacial to reach a maximum near 10 ka. It decreases gradually across the entire Holocene. Solar insolation influences $Q_{{\mathrm{div}}}$ over India (Fig. 3d). Over the BoB, $Q_{{\mathrm{div}}}$ is influenced substantially by changes in surface latent heat fluxes. These fluxes drive $Q_{{\mathrm{div}}}$ out of phase with insolation (Fig. 3e). The changes in surface latent fluxes over the BoB are influenced by the convective heating over the west equatorial Indian ocean and the middle-east (Supplementary Fig. 3). The heating of the atmospheric column over the west equatorial Indian ocean and the middle east causes a change in surface winds over the BoB^30^, and thus modulates the latent heat fluxes over the BoB. Thus, due to the opposing trends in $Q_{{\mathrm{div}}}$, during the $Q_{{\mathrm{div}}}$ dominant regime, $P-E$ over India and the BoB are out of phase.

On orbital timescale, the variations in *GMS* over India and the BoB are related to column integrated water vapor (*CWV*; units—kg $\hbox {m}^{2}$)^26^ (Supplementary Fig. 4). It has been demonstrated that *GMS* and 1/*CWV* are linearly related for the ISM^26^. This linear relation between the two quantities is also valid over the BoB on orbital timescales, albeit with different constants. This difference in constants arises on account of the different vertical structures of vertical velocity which is known to impact *GMS*^29^ (Supplementary Fig. 5). The diagnostic model attributes moisture convergence to $Q_{{\mathrm{div}}}$ and *CWV*. The dependence of *GMS* on *CWV* is more complex during periods of large millennial-scale oscillations (such as the Bølling–Allerød warming). This will be dealt with in detail in a separate section. *CWV* over both India and the BoB increases rapidly during the deglacial and reaches their highest values in the early Holocene (Fig. 3c). Across the Holocene, *CWV* is relatively stable and shows less variations. *CWV* and hence *GMS*, over India and the BoB vary in synchrony (Fig. 3b, c). Hence, during the *GMS* dominant regime, precipitation over India and the BoB show similar variations. The transition from the *GMS* dominant regime to $Q_{{\mathrm{div}}}$ dominant regime is not abrupt. Towards the end of deglacial, the influence of $Q_{{\mathrm{div}}}$ increases steadily. Thus, over the period 15–12 ka, precipitation over the BoB decreases in spite of an increase in insolation during this period.

### Effect of individual forcings

**Figure 4:**
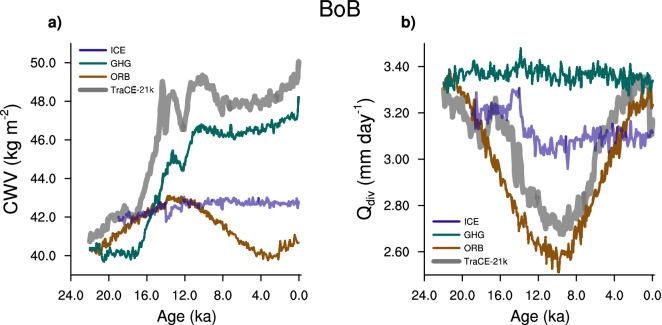
Influence of individual forcings on *CWV* and $Q_{{\mathrm{div}}}$. Time series of (**a**) column integrated water vapor (*CWV*), and (**b**) net energy flux into the atmosphere ($Q_{{\mathrm{div}}}$; top $+$ bottom) over the Bay of Bengal from the TraCE-21k (grey), orbital only simulation—ORB (in brown), greenhouse gas only simulation—GHG (in green), ice sheet only simulation—ICE (in violet).

To elucidate the effect of individual forcings we have used the transient simulations where only one of the forcing is allowed to vary (ORB: Orbital only, GHG: Greenhouse gas only, ICE: Ice sheets only, while all other boundary conditions remain fixed at their LGM values). Most of the variations in *CWV* are driven by greenhouse gas forcing (Fig. 4a). Rising greenhouse gas concentrations drive an increase in SST, and thus *CWV* (Supplementary Fig. 7). The variations in orbital forcing alone impacts $Q_{{\mathrm{div}}}$ (Fig. 4b). Hence, we focus on the ORB and GHG simulations for further analysis.

**Figure 5:**
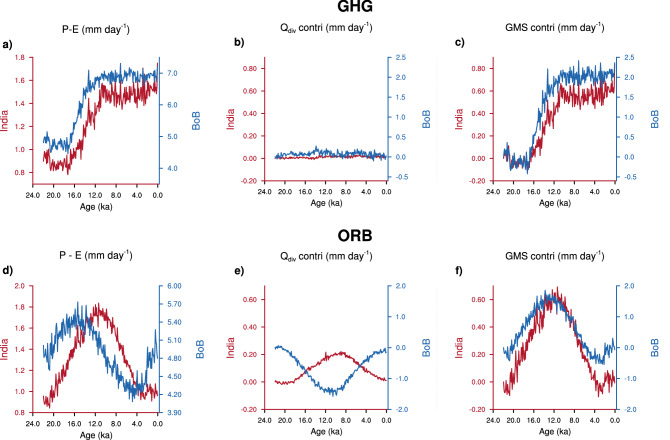
Impact of different forcings on the phase of $P-E$ between India and BoB. The time series of (**a**, **d**) $P-E$, (**b**, **e**) contribution of net energy flux into the atmosphere ($Q_{{\mathrm{div}}}$; top $+$ bottom) to change in $P-E$, and, (**c**, **f**) contribution of *GMS* to change in $P-E$. These quantities are shown in red for India, and blue for the Bay of Bengal. (**a**, **b**), and (**c**) are from the greenhouse gas only simulation (GHG), whereas, (**d**, **e**), and (**f**) are from the orbital only simulation (ORB).

In the GHG simulation, only greenhouse gas concentrations evolve, while ice sheets and orbital configuration remain fixed at their values at $\sim$ 22 ka. $P-E$ shows similar variations over both India and the BoB (Fig. 5a). Almost all of these variations in $P-E$ are explained by the variations in *GMS* (and hence *CWV*), whereas, $Q_{{\mathrm{div}}}$ does not influence $P-E$ (Fig. 5b, c). On the other hand, variations in $Q_{{\mathrm{div}}}$ make a minor contribution in the ORB simulation, and the variations in $P-E$ are mainly related to those in *GMS* (Fig. 5e, f). This leads to a small shift in the phase between $P-E$ over India and the BoB (Fig. 5d). This response is different from what occurs in the Holocene of the full TraCE-21k, where $P-E$ over India and the BoB are out of phase. Like in the ORB simulation, precession is the dominant forcing in the Holocene of TraCE-21k, but the climate is warmer. The sensitivity of *GMS* to variations in *CWV* is weak in a warm climate (Supplementary Fig. 6). Thus, the contribution of *CWV* to changes in $P-E$ is small during the warmer Holocene of TraCE-21k, and variations in $P-E$ are driven almost entirely by those in $Q_{{\mathrm{div}}}$.

To summarize, greenhouse gas forcing has a dominant influence on *CWV*, and orbital forcing modulates $Q_{{\mathrm{div}}}$. The phase shift between $P-E$ over India and the BoB depends on the relative contribution of $Q_{{\mathrm{div}}}$. When $Q_{{\mathrm{div}}}$ does not vary (e.g. GHG simulation) $P-E$ over India and the BoB are in phase, and during periods when $Q_{{\mathrm{div}}}$ is the dominant driver of $P-E$, it is out of phase between India and the BoB (e.g. Holocene of TraCE-21k).

### Centennial-to-millennial scale variations

In this section, we investigate the influence of millennial-scale climate oscillations that occurred during the deglacial on $P-E$ over the BoB. These oscillations are mainly related to the strengthening/weakening of the AMOC. The precipitation over the BoB shows the largest fluctuations corresponding to the period between 14 and 12 ka. During this period there was an abrupt warming (the Bølling–Allerød warming), followed by a cold period known as the Younger dryas (12.8–11.5 ka). During the Bølling–Allerød warming atmospheric $\hbox {CO}_{2}$ concentrations rose by 20–35 ppm within a span of 200 years^31^. Even though this rate is smaller than the anthropogenic $\hbox {CO}_{2}$ emission rate over the last 50 years, an analysis of this period can provide insights into the transient response of the climate system to greenhouse gas forcing. As noted in the earlier section, the relation between *GMS* and *CWV* is complex during these periods, hence, we propose a new model for *GMS*. In a two-layer atmosphere with weak horizontal gradients, *GMS* reduces to:

2 $$\begin{aligned} GMS = \frac{MSE_{t} - MSE_{b}}{-L_{v}\times (q_{t} - q_{b})} \end{aligned}$$

**Figure 6:**
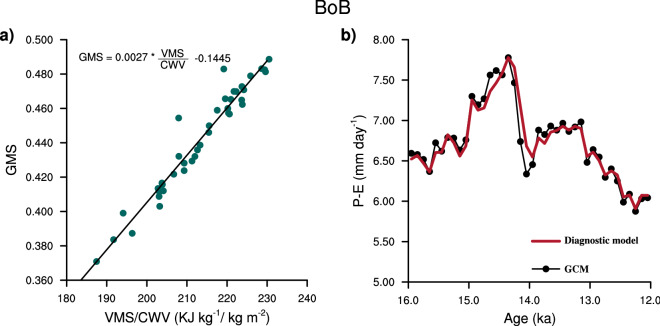
*GMS* as a function of *VMS* and *CWV*. (**a**) Scatter plot of *GMS* and the ratio of vertical moist stability (*VMS*) with column integrated water vapor (*CWV*) over the Bay of Bengal. (**b**) time series of moisture convergence ($P-E$) from the TraCE-21k (black) and the diagnostic model (red). The period between 16 and 12 ka was considered for these figures.

Here *MSE* is the moist static energy (units—KJ $\hbox {kg}^{-1}$) and *q* is the specific humidity (units—kg $\hbox {kg}^{-1}$). Subscripts ‘t’ and ‘b’ refer to integrals in the upper and lower troposphere, respectively. The numerator is a measure of vertical energy stratification and has been referred to as the vertical moist stability (*VMS*) in the previous literature^32,33^. For simplicity, we represent *VMS* as a difference between *MSE* (200 hPa) and *MSE* (800 hPa) (using the average *MSE* in the upper and lower atmosphere does not alter any of our conclusions). Since $q_{t}$ is usually very small, the denominator can be expressed as a function of *CWV*. The new simple model for *GMS* is (Fig. 6a):

3 $$\begin{aligned} GMS = 0.0027\times \frac{VMS}{CWV} - 0.1445 \end{aligned}$$

and therefore,

4 $$\begin{aligned} P - E = \frac{Q_{{\mathrm{div}}}}{ \frac{0.0027\times VMS}{ CWV } - 0.1445 } \end{aligned}$$

This equation underlines the two effects of moisture on precipitation viz., as a source of moisture (moisture availability) and as a source of buoyancy (higher water vapor increases the moist static energy in the lower atmosphere and thus lowers the *VMS*). The diagnostic model encapsulates the non-linear impact of water vapor on precipitation. The formulation of *GMS* suggested previously^26^ did not include *VMS*. This assumption works well over India, but not over the BoB at millennial timescales. This is due to the different profiles of vertical velocity over India and the BoB. Over India, the vertical velocity is bottom-heavy and the maxima of vertical velocity coincides with the minima in *MSE* (Supplementary Fig. 5). This leads to smaller variations in the vertical advection of *MSE*, and hence, a weaker dependence of *GMS* on *VMS*. On the other hand, over the BoB the vertical velocity is top-heavy resulting in large vertical advection of *MSE*. This difference in the vertical advection of *MSE* results in a stronger dependence of *GMS* on *VMS*. Therefore, during periods of abrupt climate change (such as the Bølling–Allerød) when the variations in *VMS* are large (Supplementary Fig. 8), *GMS* is influenced by *VMS* over the BoB. The new diagnostic model accounts for the variations in *VMS* and captures the fluctuations related to millennial-scale oscillations (Fig. 6b).

**Figure 7:**
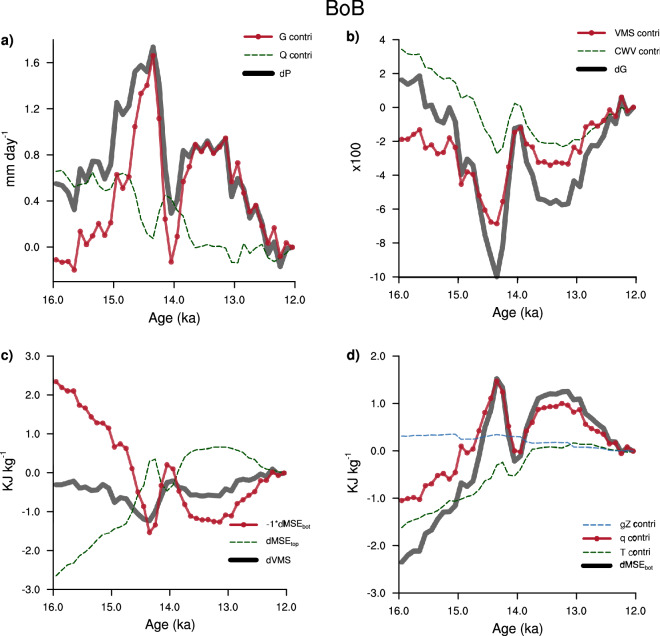
Role of low-level water vapor in driving millennial-scale oscillations. Time series of (**a**) the change in moisture convergence ($P-E$) over the Bay of Bengal (in black), contributions from gross moist stability (*GMS*; in red), and net energy flux into the atmosphere ($Q_{{\mathrm{div}}}$; in green). (**b**) the change in *GMS* (in black), contribution from vertical moist stability (*VMS*; in red), and column integrated water vapor (*CWV*; in green), (**c**) the change in moist static energy of the upper atmosphere ($MSE_{{\mathrm{top}}}$; in green), moist static energy of the lower atmosphere ($MSE_{{\mathrm{bot}}}$; in red), and *VMS* (in black). (**d**) the change in $MSE_{{\mathrm{bot}}}$ (black) and its components viz., potential energy (*gZ* in blue), internal energy ($C_{p}T$; in green), and moist energy ($L_{v}q$; in red). The century at 12 ka is chosen as the reference climate for this analysis. In (**b**), all variables are multiplied by a factor of 100.

Using the new diagnostics for moisture convergence over the BoB, we have unraveled the sequence of events that leads to abrupt changes in moisture convergence (Fig. 7). Figure 7a shows a decomposition of moisture convergence into contributions from *GMS* and $Q_{{\mathrm{div}}}$. *GMS* is dominant during the Bølling–Allerød. The variations of *GMS* during this period are related to the changes in *VMS* (Fig. 7b). *VMS* is nearly a constant through most of the deglacial, and the fluctuations in *VMS* are pronounced only during the Bølling–Allerød and the Younger Dryas. Examining *VMS* during this period suggests that the *MSE* of the lower atmosphere drives the changes in *VMS* (Fig. 7c). Moisture contributes the most to the deviations in *MSE* of the lower troposphere (Fig.  7d), which is in turn driven by changes in SST (Supplementary Fig. 7). This result concurs with the previous studies, that have shown that during the Bølling–Allerød warming and the Younger dryas cooling, SST variations in the North Atlantic affect the SST in the Indian Ocean^34,35^. The ensuing fluctuations in monsoon winds were then associated with the strengthening and weakening of the monsoon during this period^35^. Our diagnostic model, however, attributes the monsoon response to the variations in moisture.

To summarize, during periods of abrupt climate change like the Bølling–Allerød, alterations in precipitation over the BoB result from the influence of water vapor on the vertical stability of the atmosphere. The changes in SST drives the changes in water vapor.

## Discussion and conclusions

**Figure 8:**
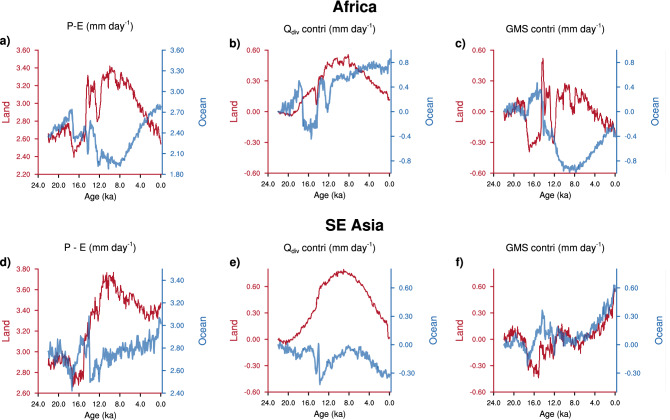
Analysis of other domains. The time series of (**a**, **d**) $P-E$, (**b**, **e**) contribution of net energy flux into the atmosphere ($Q_{{\mathrm{div}}}$; sum of top and bottom fluxes) to change in $P-E$, and, (**c**, **f**) contribution of *GMS* to change in $P-E$. These quantities are shown in red for land, and blue for ocean. (**a**, **b**), and (**c**) are from the domain covering the West African monsoon (0$^\circ$– 20$^\circ$ N and 40$^\circ$W–20$^\circ$ E; land only grids) and the adjacent Atlantic ocean (0$^\circ$–20$^\circ$ N and 40$^\circ$ W–20$^\circ$ E; ocean only grids), whereas, (**d**, **e**), and (**f**) are for the southeast Asia (0$^\circ$–30$^\circ$ N and 95$^\circ$–120$^\circ$ E; land only grids) and the adjacent Northwestern Pacific (0$^\circ$–20$^\circ$ N and 110$^\circ$–180$^\circ$ E; ocean only grids).

Terrestrial proxies for precipitation suggest a spatially coherent response across the northern monsoons. Recently, it has been shown that the intensification and meridional shifts in ITCZ are sufficient to explain most of the variations in these terrestrial proxies^4^. This cannot explain, however, the opposite response of precipitation over oceans and lands, within the same latitude band (Figs. 1a, b, 8a, d). The contribution of surface energy fluxes over the oceans drives the opposite response. This highlights the fact that the net energy flux into the atmosphere (top $+$ bottom) is the relevant quantity rather than the solar insolation. This has implications for interpretations of proxies over oceans. Recent studies suggest a phase lag of $\sim$ 9 Kyrs for precipitation in the BoB with respect to local summer insolation^36^. Our results show that while this is true, precipitation over the BoB is in sync with the net energy flux into the atmosphere over the BoB during the Holocene.

Analysis of other monsoon domains suggests that even though the co-evolution of precipitation over land and ocean is similar to that in the Indian subcontinent (Fig. 8a, d), the mechanisms are region-specific. For example, the opposite trends in $P-E$ over West Africa and the adjacent Atlantic ocean during the Holocene is due to nearly equal contributions from *GMS* and $Q_{{\mathrm{div}}}$ over both land and ocean (Fig. 8b, c). Over Southeast Asia (land grids only) the decline in $P-E$ across the Holocene is due to the decrease in $Q_{{\mathrm{div}}}$, whereas, the increase in $P-E$ over the adjacent Northwest Pacific is driven by *GMS* (Fig. 8e, f). Further studies are necessary to decipher a detailed mechanism over these regions.

In this study, we have explored in-depth, the evolution of precipitation over India in relation to that over the Bay of Bengal, using a fully coupled transient simulation. We have shown that during cold periods (like LGM and deglacial), water vapor exerts greater control over the Indian monsoon. Since water vapor has similar trends over land and ocean on orbital timescales, ITCZ has a uniform response during the cold, water vapor driven periods. The influence of net energy flux is dominant during warm periods (like the Holocene). Thus, driving a differential response of ITCZ over land and ocean. The speleothem proxy for precipitation over the BoB attests to the opposite response of precipitation over oceans predicted by climate models (Supplementary Fig. 1). There is a need to unravel longer precipitation records over the oceans. Our results highlight the fact that global monsoons should not be treated as a homogeneous entity driven by solar insolation over both land and ocean. Apart from the contribution of surface energy fluxes, the vertical profile of vertical velocity over oceans is different than that over the land, leading to differences in the export of moist static energy.

In our previous work, we showed that it is enough to consider net energy flux and water vapor to diagnose changes in the Indian monsoon^26^. We have now demonstrated that vertical energy stratification needs to be taken into account along with net energy flux and water vapor to explain the millennial-scale oscillations over oceans. We find that during these periods water vapor modulates precipitation through its influence on vertical stability. The current anthropogenic global warming poses an even larger abrupt forcing. The diagnostic model suggested in this study has the potential for deciphering a plausible mechanism for monsoon response to global warming in the twenty-first century over both land and ocean.

## Methods

### The transient simulation

The fully coupled model Community Climate System Model version 3 (CCSM3)^37^ was used to simulate the climate since the last glacial maximum (21 ka). This simulation is known as the Transient Climate Evolution of the last 21,000 years (TraCE– 21k). The model is forced with realistic transient forcings (variations in Earth’s orbit, greenhouse gases, ice sheets, and meltwater fluxes)^14^. This simulation captures the key climatic features over the last 22,000 years^13,38^. The long term trends in the African and Asian monsoons are reproduced quite well^24, 26,39,40^. There is, however, a dry bias^26,41^. Over the domain of our interest (Fig. 1b) Jun–Jul–Aug precipitation in the present day climate (1950–1990 A.D.) is underestimated by about 33% over India^26^ and overestimated by about 4% over the Bay of Bengal. This is well within the CMIP5 model spread (Supplementary Fig. 2). Since, our objective is to explain the cause of long term variations in monsoons, these biases do not affect our results. To understand the role of individual forcings, simulations were carried out where only one forcing was specified (ORB—orbital only, GHG—greenhouse gas only, ICE—ice sheet only, and MWF—meltwater flux). We have used centennially averaged data in all of our analyses. Therefore, the entire period of 22,000 years is divided into 220 continuous but non-overlapping centuries.

### Diagnostic methdology

The energetic framework regards monsoon as an energetically direct circulation. Precipitation is related to moisture convergence, which depends on circulation driven by the conservation of energy. The diagnostic model that results from using the vertically integrated moisture and moist static energy (*MSE*) ascribes precipitation to energy fluxes and vertical stability of the atmosphere^28,29^. Taking the ratio of vertically integrated moist static energy and moisture equations (after multiplying the moisture equation by the latent heat of vaporization), we get:

5 $$\begin{aligned} P - E= & {} \frac{Q_{div}}{ - \frac{ \left\langle \nabla \cdot m\vec {U} \right\rangle + \left\langle \frac{\partial m\omega }{\partial p} \right\rangle }{ L \left\langle \nabla \cdot q\vec {U} \right\rangle + L \left\langle \frac{\partial q\omega }{\partial p} \right\rangle } } = \frac{Q_{div}}{GMS} \end{aligned}$$

6 $$\begin{aligned} GMS= & {} -\frac{ \left\langle \nabla \cdot m\vec {U} \right\rangle + \left\langle \frac{\partial m\omega }{\partial p} \right\rangle }{ L \left\langle \nabla \cdot q\vec {U} \right\rangle + L \left\langle \frac{\partial q\omega }{\partial p} \right\rangle } = \frac{Q_{div}}{P - E} \end{aligned}$$

where the angle brackets ( $\left\langle . \right\rangle$) represent vertical integrals. All the variables have been described in Table 1. A detailed derivation can be found in previous studies^8,27–29^.

**Table 1:** Definition of variables.

Variable	Description
*m*	Moist static energy (J $\hbox {kg}^{-1}$), which is the sum of internal energy, potential energy, and moist energy ($C_{p}T + gZ + L_{v}q$)
$\omega$	Vertical component of velocity (Pa $\hbox {s}^{-1}$)
$\mathbf {U}$	Horizontal velocity ($u\mathbf {i} + v\mathbf {j}$) (m $\hbox {s}^{-1}$)
$Q_{{\mathrm{div}}}$	Net energy flux into the atmosphere (in mm $\hbox {day}^{-1}$; taking the latent heat of vaporization of water as $2.501 \times10^{6}$ J $\hbox {kg}^{-1}$ we get 1 mm $\hbox {day}^{-1}$ = 28.95 W $\hbox {m}^{-2}$). It is given by: $Q_{{\mathrm{div}}} = S(1 - \alpha ) - OLR + LHF + SHF + \hbox {Net}\_\hbox {sfc}\_\hbox {radiative}\_\hbox {flux}$, where *S* is insolation, $\alpha$ is the shortwave reflectivity at the top of the atmosphere, and *OLR* is the outgoing longwave radiation, *LHF*, and *SHF* are the surface latent and sensible heat fluxes, respectively
*q*	Specific humidity (kg $\hbox {kg}^{-1}$)
*P*	Precipitation rate (mm $\hbox {day}^{-1}$)
*E*	Evaporation rate (mm $\hbox {day}^{-1}$)

This table describes all the variables used in the “Methods” section.

### Calculation of relative contribution of GMS and energy

Equation 5 is shown below for a reference climate by using the subscript (0).

7 $$\begin{aligned} (P - E)_{0} = \frac{Q_{{\mathrm{div0}}}}{GMS_{0}} \end{aligned}$$

Taking the ratio of Eqs. 5 and 7 gives the following:

8 $$\begin{aligned} \frac{(P - E)}{(P-E)_{0}} = \frac{Q_{{\mathrm{div}}}}{Q_{{\mathrm{div0}}}}\times \frac{GMS_{0}}{GMS} \end{aligned}$$

This equation is used in Fig. 2 to show the evolution of relative dominance of $Q_{{\mathrm{div}}}$ and *GMS* over the last 22,000 years.

To calculate the contribution of $Q_{{\mathrm{div}}}$ and *GMS* in mm $\hbox {day}^{-1}$, to a change in $P-E$ between two climates, we use the following equation, which was derived earlier^8^:

9 $$\begin{aligned} \underbrace{ \Delta (P-E) }_{\hbox {Change in P-E}} \; = \underbrace{\frac{\frac{\Delta Q}{Q}}{1 + \frac{\Delta G}{G}}(P-E)}_{\hbox {Contribution from }Q_{div}} + \underbrace{\frac{-\frac{\Delta G}{G}}{1 + \frac{\Delta G}{G}}(P-E)}_{\hbox {Contribution from GMS}} \end{aligned}$$

*Q* and *G* are short-forms used in place of $Q_{{\mathrm{div}}}$ and *GMS*, respectively. $\Delta$ represents the perturbation of the climate of interest with respect to a reference climate (we have taken the climate at 22 ka as a reference in Figs. 5, 8). The term on the LHS is the change in $P-E$. The first term on the RHS gives the contribution from $Q_{{\mathrm{div}}}$, whereas the second term gives the contribution from *GMS*.

### Calculation of relative contribution of VMS and water vapor

To quantify the contribution of *VMS* and *CWV* to changes in *GMS* we do the following. Consider the equation for *GMS* for a reference climate:

10 $$\begin{aligned} GMS_{0} = 0.0027\times \frac{VMS_{0}}{CWV_{0}} - 0.1445 \end{aligned}$$

Here subscript (0) represents the reference climate. Taking a difference between the above equation written for a climate of interest and the reference climate:

11 $$\begin{aligned} \Delta GMS = 0.0027\times \left( \frac{VMS}{CWV} - \frac{VMS_{0}}{CWV_{0}} \right) \end{aligned}$$

where $\Delta$ is the perturbation ($\Delta () = () - ()_{0}$). This can be rearranged to get:

12 $$\begin{aligned} \underbrace{ \Delta GMS}_{\hbox {Change in GMS}} = \underbrace{0.0027\times \frac{\Delta VMS}{CWV_{0}} }_{\hbox {Contribution from VMS}} - \underbrace{0.0027\times \frac{VMS_{0}\times \Delta CWV}{CWV_{0}\times CWV} }_{\hbox {Contribution from CWV}} \end{aligned}$$

## Supplementary information

Supplementary information.

## Data Availability

The $\delta ^{18}$O data from the core KL-126 used in the Supplementary Fig. 1 are obtained from (https://doi.pangaea.de/10.1594/PANGAEA.735053). The $\delta ^{18}$O data from a speleothem in the Baratang cave was provided by the corresponding author of Laskar et al.^9^. The TraCE-21k dataset analyzed in this study, can be downloaded from the Earth System Grid (National Center for Atmospheric Research) (https://www.earthsystemgrid.org/project/trace.html). The GPCP Precipitation data and the NCEP reanalysis 1 data used in this study were provided by the NOAA/OAR/ESRL PSD, Boulder, Colorado, USA, from their Web site at https://www.esrl.noaa.gov/psd/. The CMIP5 data sets are publicly available at https://esgf-data.dkrz.de/search/cmip5-dkrz/.

## Code availability

The codes are available from the corresponding author C.J., on request.
