# Supplementary material for: Different precipitation response over land and ocean to orbital and greenhouse gas forcing
Source: Sci Rep. 2020 Jul 17;10:11891. doi: 10.1038/s41598-020-68346-y (PMC7367857; doi:10.1038/s41598-020-68346-y)
Supplement: Supplementary file 1 — Supplementary information. [file 41598_2020_68346_MOESM1_ESM.pdf]

Supplementary information

Different precipitation response over land and ocean to orbital and greenhouse gas forcing

Chetankumar Jalihal[1,2,*], Jayaraman Srinivasan[2] & Arindam Chakraborty[1,2]

[1]*Centre for Atmospheric and Oceanic Sciences, Indian Institute of Science, Bangalore, 560012, India.*

[2]*Divecha Centre for Climate Change, Indian Institute of Science, Bangalore, 560012, India.*

**email: jalihal@iisc.ac.in*

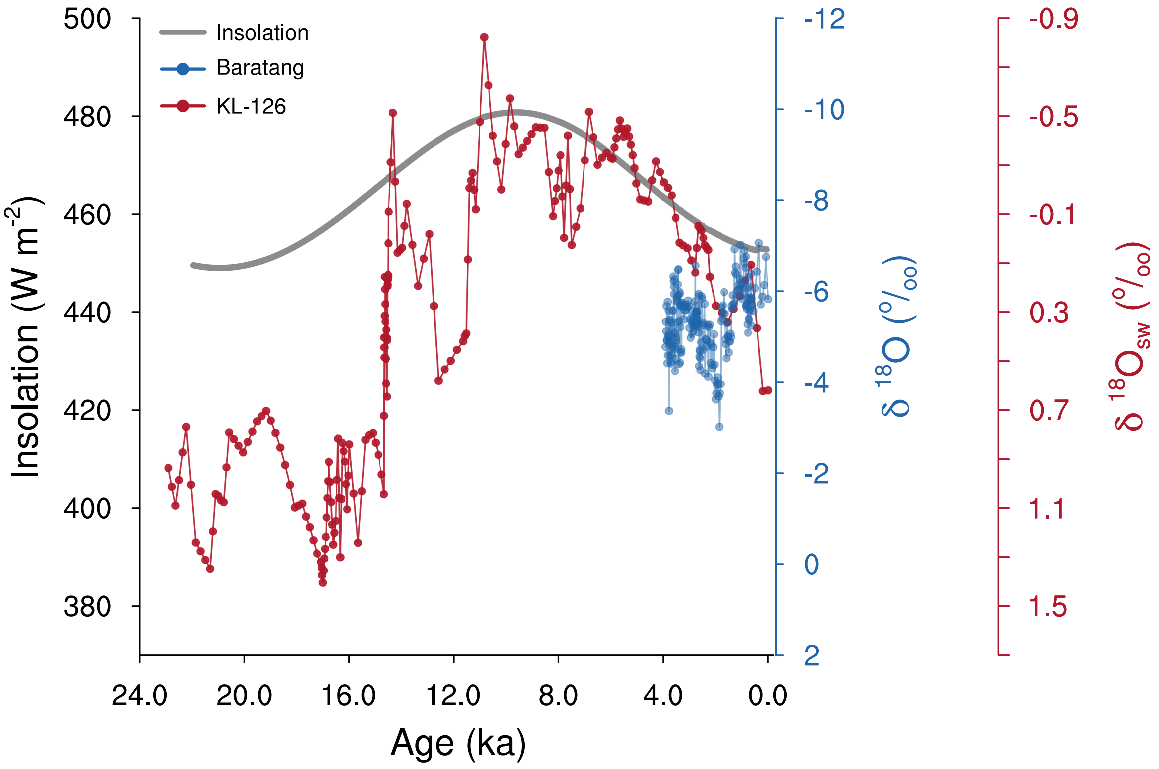

Supplementary Figure 1: Temporal evolution of precipitation over India and the Bay of Bengal. The time series of summer insolation over India (10°–29°N and 70°–95°E)(Jun-Jul-Aug mean) in grey, $\delta^{18}O_{sw}$ ($\delta^{18}O$ of seawater) in red from the sediment core KL-126[1], and $\delta^{18}O$ from a speleothem from the Baratang cave in the Andaman islands[2]. The $\delta^{18}O_{sw}$ from KL-126 is a proxy for sea surface salinity in the North Bay of Bengal. This region is influenced by run-off from precipitation over India. Hence, it represents the strength of the Indian summer monsoon. The $\delta^{18}O$ from the Baratang cave is an indicator of the precipitation over the Bay of Bengal.

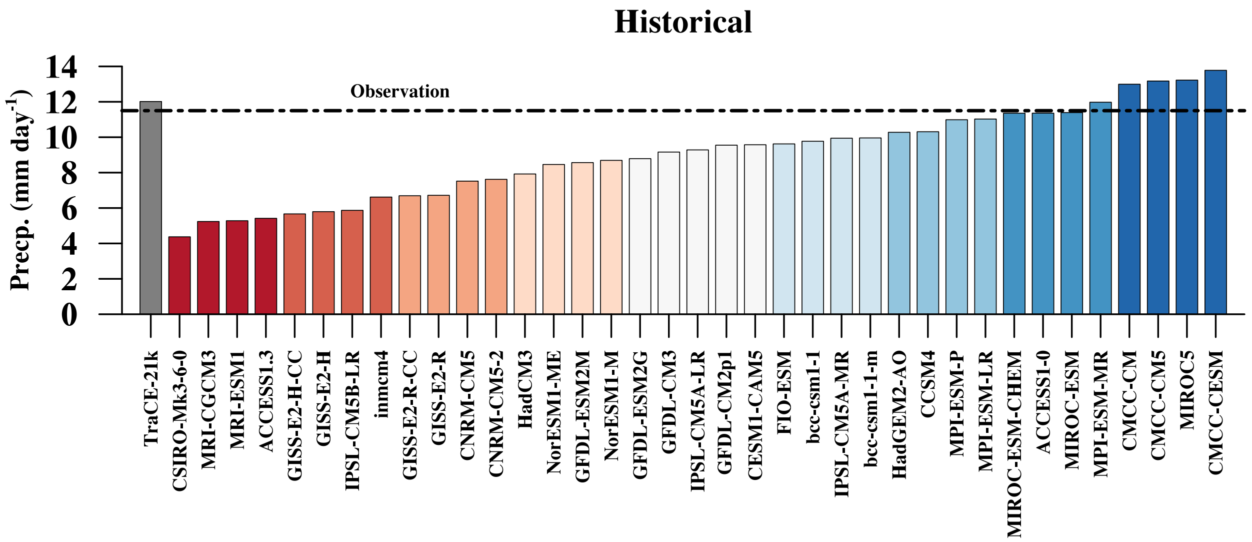

Supplementary Figure 2: Comparison of TraCE-21k with observation and CMIP5. The bar graph shows a comparison of the Jun-Jul-Aug (JJA) mean precipitation over the Bay of Bengal (9°–20°N and 85°–95°E; ocean only points) taken from the TraCE-21k with all the models that participated in the CMIP5 historical experiment. The climatology of JJA precipitation from GPCP (Global Precipitation Climatology Project) over the period (1979-2018 AD) is shown for reference. The climatology from the TraCE-21k is evaluated over the period 1800-1990 AD.

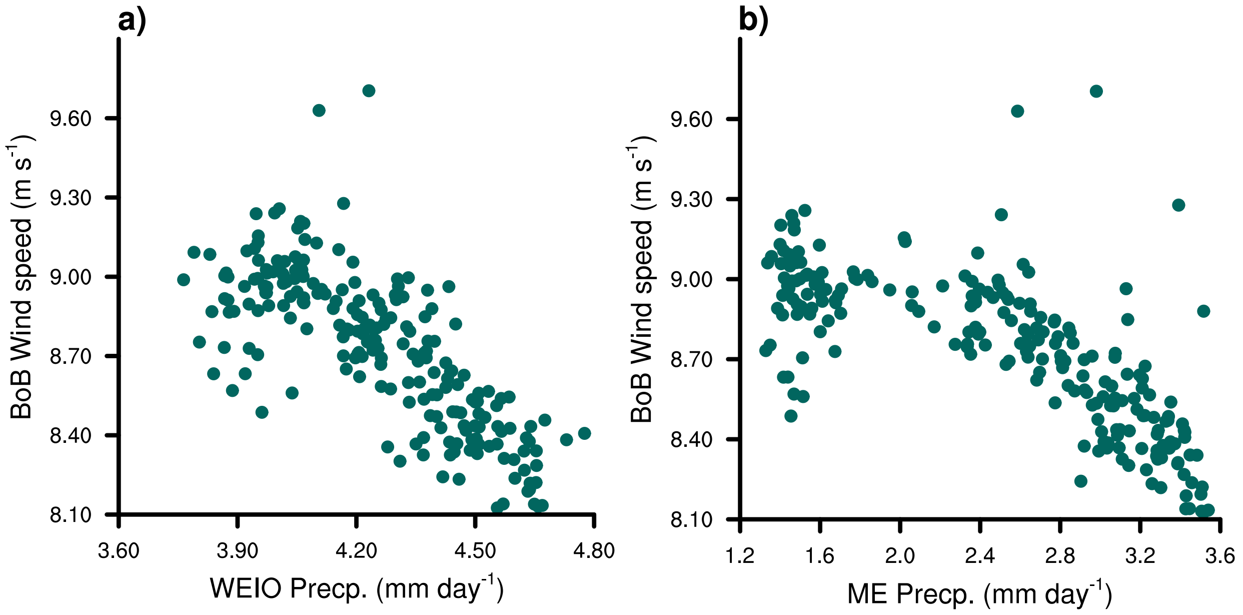

Supplementary Figure 3: Influence of precipitation to the west of the Bay of Bengal on local winds. Scatter plot of Jun-Jul-Aug (JJA) mean wind speed at 850 hPa over the Bay of Bengal (BoB), with JJA precipitation over (**a**), west equatorial Indian ocean (WEIO; 10°S–10°N and 50°–60°E), and (**b**) Middle-east (ME; 15°–25°N and 50°–60°E). The entire 22,000 years were divided into 220 non-overlapping centuries. Every filled circle is the mean over a century.

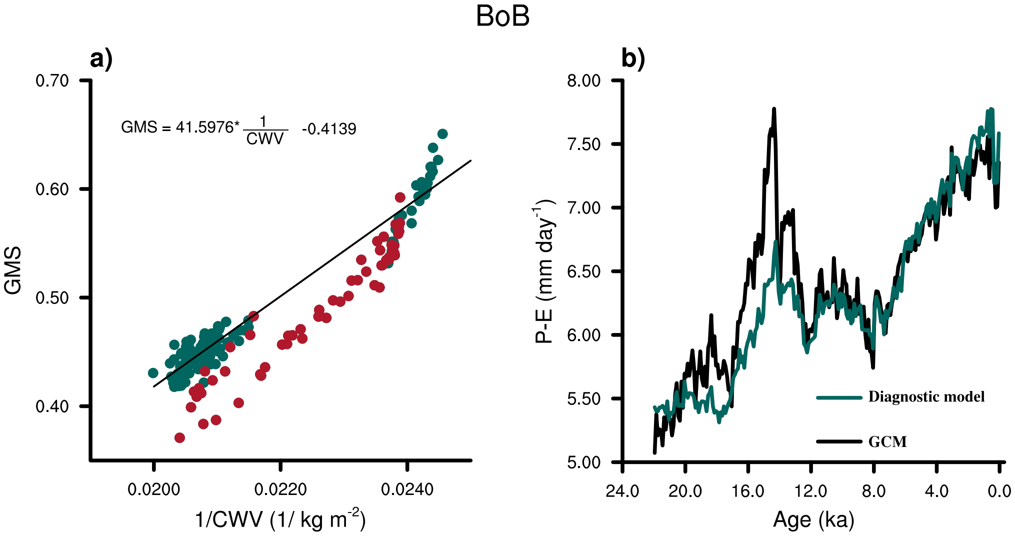

Supplementary Figure 4: Relation between gross moist stability and water vapor. (**a**), Scatter plot of Jun-Jul-Aug (JJA) mean gross moist stability (GMS) evaluated over the Bay of Bengal with column integrated water vapor (CWV) over the same region and months. The red filled circles correspond to the time period between 19 ka to 13 ka. Only the green filled circles were considered for obtaining the linear fit. (**b**) Time series of moisture convergence ($P-E$) over the Bay of Bengal from the TraCE-21k in black and from the diagnostic model in green.

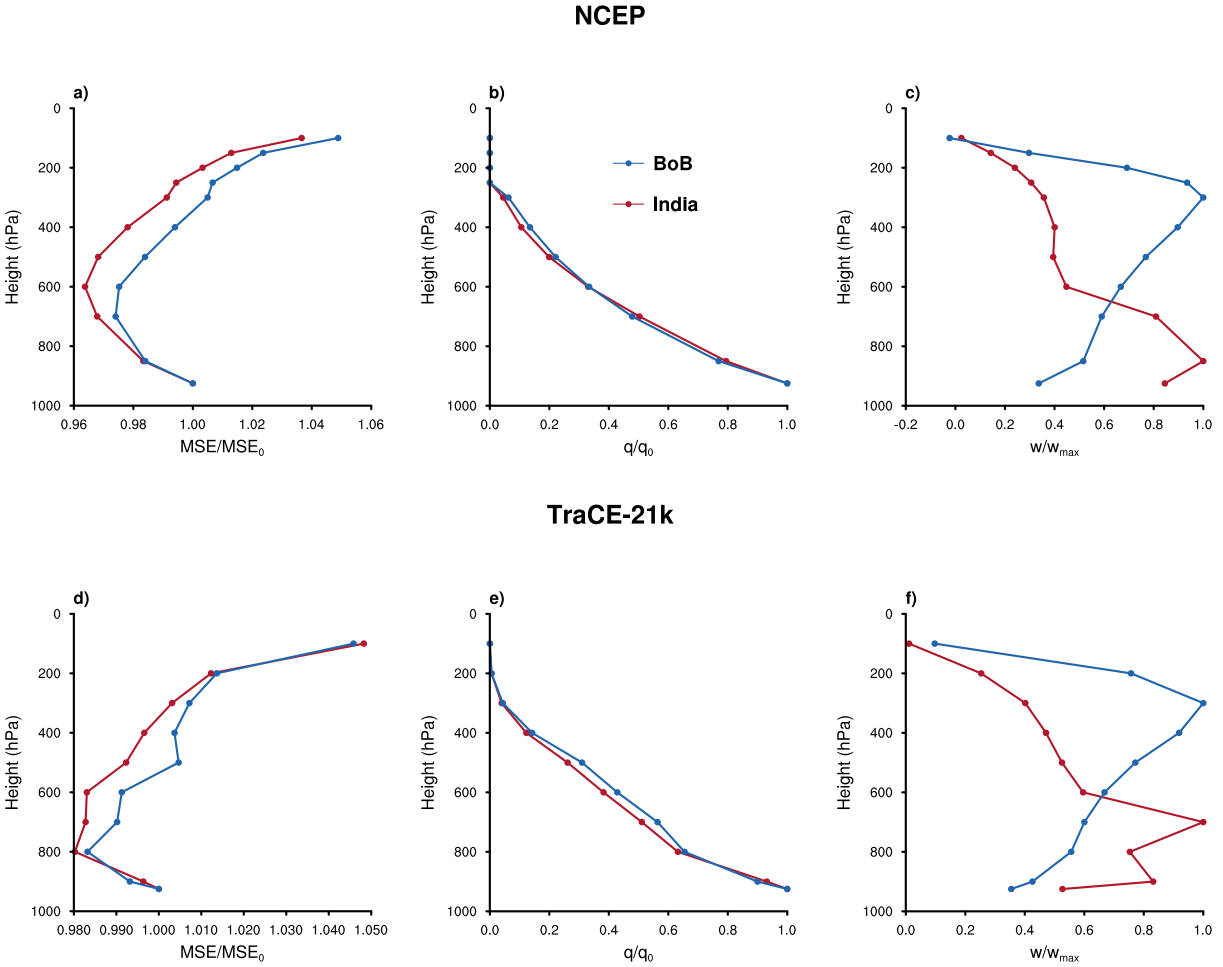

Supplementary Figure 5: Difference in vertical profiles between India and the Bay of Bengal. The climatological vertical profiles of (**a** & **d**) moist static energy (MSE) normalized by its value at the lowest level, (**b** & **e**) specific humidity (q) normalized by its value at the lowest level, and (**c** & **f**) vertical pressure velocity (ω) normalized by the lowest value of ω. The red and blue lines represent India and the Bay of Bengal (BoB). **a, b** & **c** is for the NCEP reanalysis 1 data over the period 1948-2018 AD, whereas **d, e** & **f** is for the TraCE-21k (climatology over the period 1950-1990 AD).

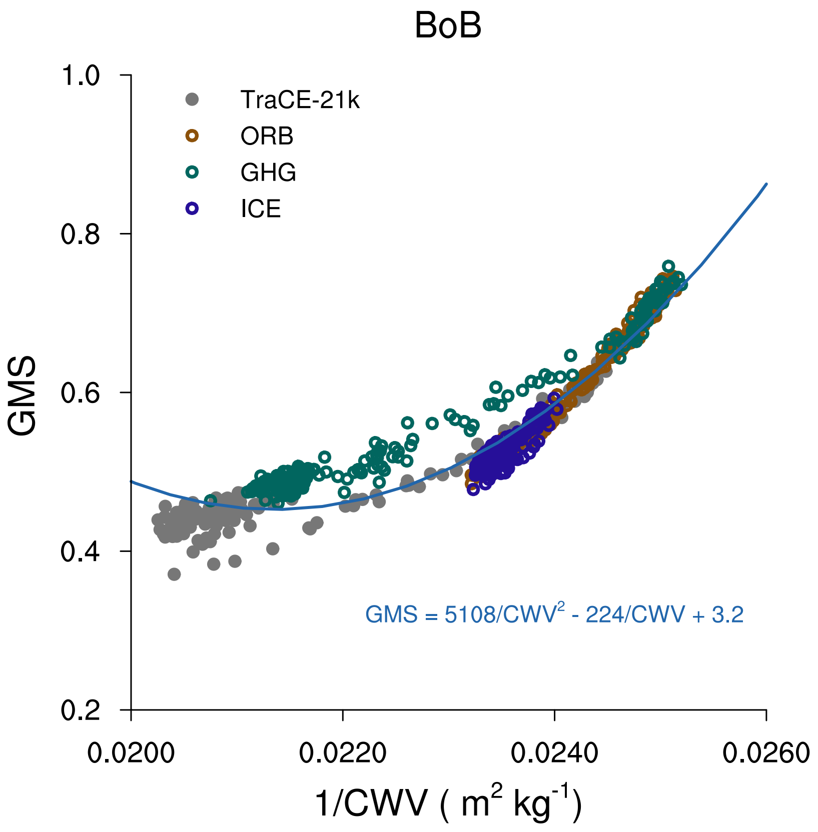

Supplementary Figure 6: Non-linear relation between gross moist stability and water vapor. Scatter plot of Jun-Jul-Aug (JJA) mean gross moist stability (GMS) over the Bay of Bengal with column integrated water vapor (CWV) over there. Grey filled circles represent the TraCE-21k simulation, whereas the open circles are from the simulations where only one of the forcings varied: ORB (orbital only; brown), GHG (greenhouse gas only; green), and ICE (ice sheet; violet). Every circle is an average over a century.

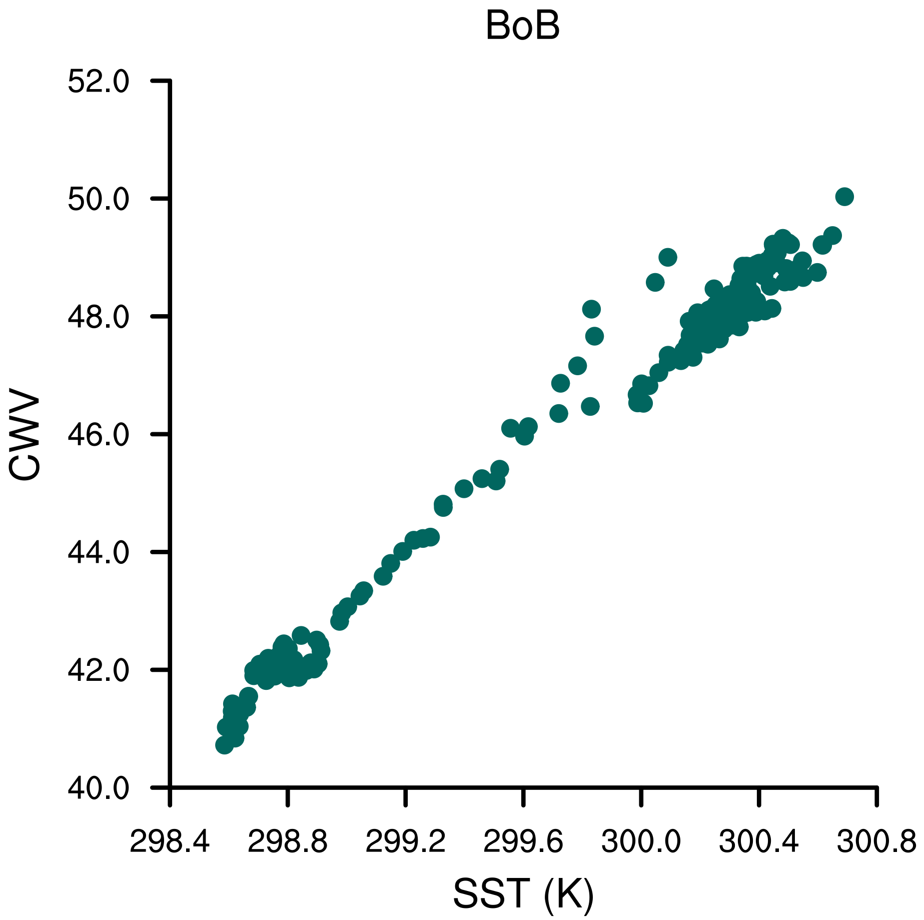

Supplementary Figure 7: Influence of sea surface temperature on water vapor. Scatter plot of Jun-Jul-Aug (JJA) mean sea surface temperature (SST) over the Bay of Bengal with column integrated water vapor (CWV) over there.

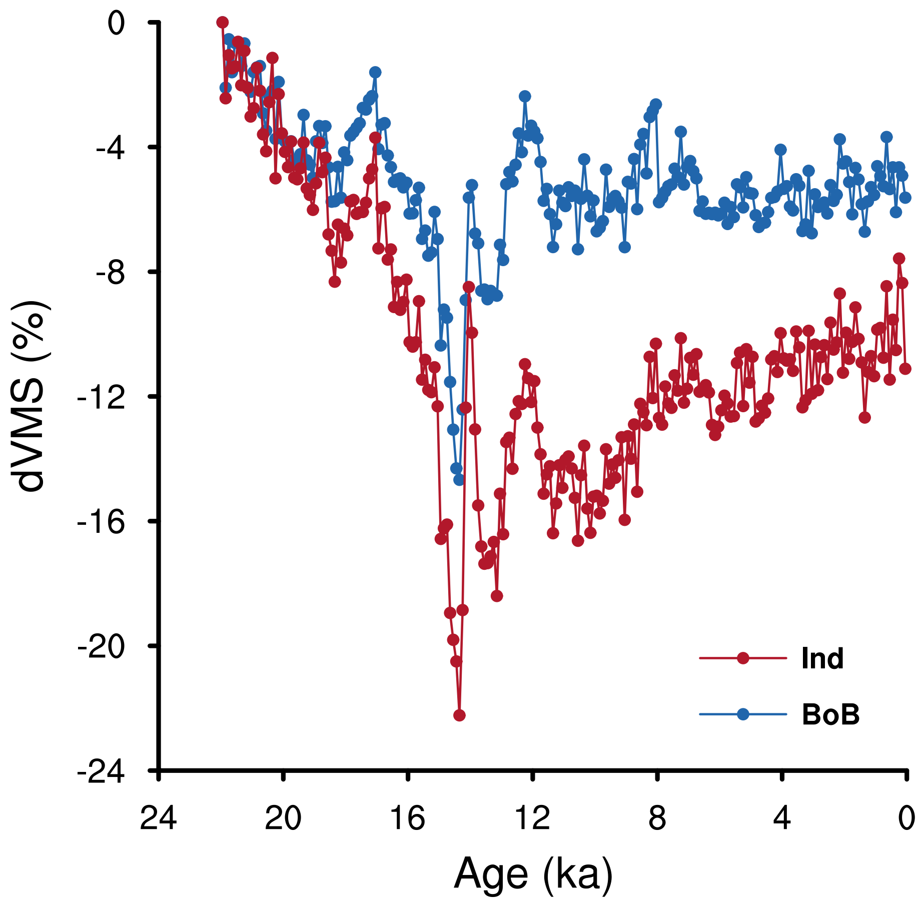

Supplementary Figure 8: Temporal variations in vertical moist stability. The time series of the percentage change in Jun-Jul-Aug (JJA) mean vertical moist stability (VMS) over India (in red), and the Bay of Bengal (in blue). The climatology over the period 22 ka to 21.9 ka is taken as a reference to evaluate the change in VMS.

Supplementary References

1. Kudrass, H., Hofmann, A., Doose, H., Emeis, K. & Erlenkeuser, H. Modulation and amplification of climatic changes in the northern hemisphere by the indian summer monsoon during the past 80 ky. *Geology* **29**, 63–66 (2001).

2. Laskar, A. H., Yadava, M., Ramesh, R., Polyak, V. & Asmerom, Y. A 4 kyr stalagmite oxygen isotopic record of the past indian summer monsoon in the andaman islands. *Geochemistry, Geophysics, Geosystems* **14**, 3555–3566 (2013).
